# Dual somatic UBA1 Met41 variants with distinct allele frequencies in VEXAS syndrome

**DOI:** 10.1016/j.ero.2026.100207

**Published:** 2026-07-03

**Authors:** Guiset Carvajal Bedoya, Marcela Ferrada, Daniela Gnecco

**Affiliations:** Department of Rheumatology, Billings Clinic, Billings, Montana, USA; University of Maryland School of Medicine, Baltimore, Maryland, USA; Pontificia Universidad Javeriana, Bogotá, Colombia

Dear Editor,

Vacuoles, E1 enzyme, X-linked, autoinflammation, somatic (VEXAS) syndrome is an adult-onset autoinflammatory disease caused by somatic mutations in *UBA1*, most commonly involving methionine-41 (Met41), and characterised by systemic inflammation and cytopenias. We described a patient with VEXAS syndrome harbouring 2 distinct somatic *UBA1* Met41 variants with markedly different variant allele frequencies (VAFs), suggesting clonal heterogeneity.

A 76-year-old man presented in May 2025 with cytopenias, 50-pound unintentional weight loss over 6 months, inflammatory arthritis involving the ankles, metatarsophalangeal joints, and hips, vasculitic rash, cough, and clinical phlebitis. He denied fevers, venous thromboembolism, or chondritis. Laboratory evaluation demonstrated leukopenia (white blood cell [WBC] count, 2.99 × 10⁹/L); anaemia (haemoglobin concentration, 8.0 g/dL); platelet count, 189 × 10⁹/L; ferritin concentration, 942 ng/Ml; erythrocyte sedimentation rate (ESR), 91 mm/h; and C-reactive protein (CRP) concentration, 2.8 mg/dL. Autoimmune serologies were negative.

Imaging revealed no lymphadenopathy and only faint ground-glass opacities in the lung bases. Bone marrow biopsy showed no malignancy or overt dysplasia; cytoplasmic vacuolisation was not reported. Given persistent systemic inflammation and cytopenias, targeted sequencing was performed and identified 2 somatic *UBA1* variants affecting codon 41: c.121A>G (p.Met41Val; VAF 66.0%) and c.122T>C (p.Met41Thr; VAF 14.9%).

A diagnosis of VEXAS syndrome was established. Treatment with prednisone (60 mg daily) resulted in rapid resolution of rash, arthritis, and cough, with improvement in leukopenia and anaemia, although platelet counts decreased modestly. Due to steroid dependence, subcutaneous tocilizumab (162 mg weekly) was initiated.

Most reported cases of VEXAS syndrome involve a single *UBA1* mutation at Met41 [[1], [2], [3]]. The presence of 2 distinct substitutions at this hotspot with discordant allele frequencies suggests a complex clonal architecture, potentially reflecting either sequential mutation acquisition within a dominant clone or the coexistence of independent subclones. The higher VAF of the Met41Val variant supports its role as the dominant clone, while the Met41Thr variant may represent a subclonal population.

This case expanded the mutational landscape of VEXAS syndrome and highlighted that clonal heterogeneity may occur at the canonical Met41 hotspot. Notably, the absence of classic features such as fever or chondritis underscores the phenotypic variability of this condition and the importance of genetic testing in older patients with unexplained cytopenias and systemic inflammation.

Further studies are needed to determine whether clonal architecture influences disease phenotype, progression, or therapeutic response in VEXAS syndrome. Table 1

**Table 1:** Summary of clinical features

Category	Findings
Age (y)/sex	76-y-old male
Initial presentation (May 2025)	Cytopenias, inflammatory arthritis, vasculitic rash, weight loss (∼50 lb/6 mo), cough, phlebitis
Constitutional symptoms	Weight loss; no fevers
Chondritis/thrombosis	Absent
Haematologic findings	WBC count, 2.99 × 10⁹/L; haemoglobin concentration, 8.0 g/dL; MCV level, 112.2 fL; platelet counts 189 × 10⁹/L; ANC level, 2.01 × 10⁹/L
Inflammatory markers	ESR rate, 91 mm/h; CRP concentration, 2.8 mg/dL; ferritin concentration, 942 ng/mL
Autoimmune serologies	ANA level and DAT test negative
Bone marrow biopsy	No malignancy or overt dysplasia; blasts not increased; vacuolisation not reported
Imaging	No lymphadenopathy; faint ground-glass opacities in lung bases
*UBA1* mutations	p.Met41Val (VAF 66.0%); p.Met41Thr (VAF 14.9%)
Diagnosis	VEXAS syndrome
Initial therapy	Prednisone 60 mg daily Prophylaxis: trimethoprim-sulfamethoxazole 800/160 mg 3 times weekly (Monday, Wednesday, and Friday) Valacyclovir 1 g daily
Response	Resolution of rash, arthritis, and cough; improvement in leukopenia and anaemia
Steroid-sparing therapy	Tocilizumab 162 mg weekly Prednisone has been tapered to 20 mg daily, with ongoing tapering by 5 mg every 3 wk
Current status (June 2026)	No active systemic disease; resolution of leukopenia and anaemia, with development of thrombocytopenia (platelet count, 80 × 10⁹/L) No infectious complications have occurred to date

ANA, antinuclear antibody; ANC, absolute neutrophil count; CRP, C-reactive protein; DAT, direct antiglobulin test; MCV, mean corpuscular volume; WBC, white blood cell; VAF, variant allele frequencies; VEXAS, vacuoles, E1 enzyme, X-linked, autoinflammation, somatic.

## CRediT authorship contribution statement

**Guiset Carvajal Bedoya:** Writing – original draft. **Marcela Ferrada:** Writing – review & editing. **Daniela Gnecco:** Writing – review & editing.

## Competing interests

Authors have no competing interests to declare.

## References

[1] Beck D.B., Ferrada M.A., Sikora K.A., Ombrello A.K., Collins J.C., Pei W. (2020). Somatic mutations in UBA1 and severe adult-onset autoinflammatory disease. N Engl J Med..

[2] Poulter J.A., Collins J.C., Cargo C., De Tute R.M., Evans P., Ospina Cardona D. (2021). Novel somatic mutations in UBA1 as a cause of VEXAS syndrome. Blood..

[3] Georgin-Lavialle S., Terrier B., Guedon A.F., Heiblig M., Comont T., Lazaro E., Lacombe (2022). Further characterization of clinical and laboratory features in VEXAS syndrome. Ann Rheum Dis.

